# Changes in Neurodegeneration and Visual Prognosis in Branch Retinal Vein Occlusion after Resolution of Macular Edema

**DOI:** 10.3390/jcm13030812

**Published:** 2024-01-31

**Authors:** Chanjoon Park, Ji Ho Lee, Young Gun Park

**Affiliations:** 1 Department of Ophthalmology and Visual Science, Seoul St. Mary’s Hospital, College of Medicine, The Catholic University of Korea, Seoul 06591, Republic of Korea; guessmyname@naver.com; 2 Catholic Institute for Visual Science, College of Medicine, The Catholic University of Korea, Seoul 06591, Republic of Korea; lee_ciel@naver.com

**Keywords:** branch retinal vein occlusion, neurodegeneration, prognosis, macular edema

## Abstract

This study aimed to examine the thicknesses of the ganglion cell layer (GCL) and peripapillary retinal nerve fiber layer (RNFL) in eyes with resolved macular edema (ME) in branch retinal vein occlusion (BRVO) and determine their relationship with visual acuity (VA). This retrospective observational case–control study included 57 eyes of BRVO patients with resolved ME after treatment. The macular GCL thickness, peripapillary RNFL thickness, and central macular thickness (CMT) measured on swept-source optical coherence tomography scans with the contralateral eyes used as controls were evaluated. The mean CMT was 270.48 ± 32.7 μm; the mean RNFL thickness was 105.46 ± 25.94 μm in BRVO eyes. Although the average RNFL thickness was decreased in BRVO eyes compared to unaffected eyes, there was no significant difference between the groups. However, the temporal and nasal RNFL thicknesses were significantly different between the groups. The mean affected quadrant had a significantly thinner GCL compared to the corresponding opposite unaffected quadrant (*p* = 0.02). Final VA was significantly correlated with nasal and middle GCL thicknesses in the affected area (*r* = −0.512, *p* = 0.003 and *r* = −0.537, *p* = 0.001, respectively); no correlation was found between the average RNFL thickness and mean CMT. The peripapillary RNFL and GCL thicknesses of the affected area were reduced in BRVO eyes compared to unaffected eyes. VA significantly correlated with nasal and middle GCL thicknesses in the affected area. Inner retinal damage occurring in patients with ME secondary to BRVO may be related to the visual prognosis.

## 1. Introduction

Branch retinal vein occlusion (BRVO) is a common retinal vascular disorder that causes retinal hemorrhages, macular edema (ME), and retinal ischemia. Most BRVOs occur because a thickened retinal arteriole wall compresses the narrowing vein [1,2]. Decreased retinal perfusion due to venous thrombosis induces the upregulation of vascular endothelial growth factor (VEGF), thereby causing a breakdown of the blood–retinal barrier. In BRVO, especially, ischemia of the retina can cause atrophy of the retinal nerve fiber layer (RNFL). In the acute phase of BRVO, pressure increases in the affected vessels and flame-shaped hemorrhage can be seen at the RNFL. This is often associated with macular edema and serous retinal detachment. RNFL thickness increases due to edema of the ischemic area. However, after the edema resolves over time, the RNFL thickness decreases gradually.

With the advent of intravitreal anti-vascular endothelial growth factor (anti-VEGF) agents, outcomes of eyes with BRVO have improved significantly. However, ME in BRVO eyes often recurs and needs frequent injections. Following repeated administration of anti-VEGF and corticosteroids, such as dexamethasone, ME resolves, and retinal thickness returns to the normal level before the acute event of BRVO. However, retinal thinning beyond the normal range is sometimes observed after treatment and leads to a deterioration of function [3,4]. In such cases, the possible mechanisms for persistent visual impairment including potential abnormalities in the retinal microvasculature cannot be determined by measurements of central macular thickness (CMT) with conventional optical coherence tomography (OCT).

Changes in the outer retina, such as in the photoreceptors, the external limiting membrane (ELM), or the ellipsoid zone (EZ), are strongly correlated with visual acuity (VA) in BRVO [5,6]. Some researchers reported that retinal ganglion cell (RGC) neurons are vulnerable to microvascular ischemia in diabetic retinopathy [7]. This RGC damage may be progressive with chronic inflammation and it might have an effect on neurodegeneration. However, fewer studies have assessed the changes in the inner retina, particularly the ganglion cell layer (GCL). 

Recent studies have shown that GCL thickness measured using OCT is useful for assessing glaucoma, optic nerve disease, and macular disease [8,9]. Swept-source OCT (SS-OCT; DRI Triton OCT, Topcon, Tokyo, Japan) is known for its ability to capture a wide 12 × 9 mm^2^ area in a single scan, attributed to its faster scanning speed and longer wavelength penetration compared to spectral domain (SD)-OCT [10,11]. The wide-field map captured with SS-OCT is superior to the conventional thickness and deviation maps obtained by SD-OCT. The wide-field GCL+ maps and the RNFL map have been used to evaluate RNFL/GCL thickness in various retinal diseases [12,13].

Previous studies have focused on investigating RNFL, i.e., a thinner RNFL following BRVO [14]. The RNFL contains RGC axons, and the GCL consists of the RGCs and displaced amacrine cells [15,16]. RGCs are responsible for propagation of visual information to the brain through axonal transport. Furthermore, experimental studies have demonstrated that RGCs are impaired in BRVO, suggesting that BRVO also has a significant neuronal factor causing its pathogenesis in relation to microvascular changes [17].

In the present study, we investigated the quantitative changes in the inner retina based on OCT after resolution of ME. We aimed to examine the thicknesses of the GCL and peripapillary RNFL and determine their relationship with VA in eyes with resolved ME in BRVO.

## 2. Materials and Methods

### 2.1. Ethics Statement

All procedures were conducted in accordance with the Declaration of Helsinki (1964) and its later amendments. This retrospective study was conducted using data from medical records. Approval was obtained from the Institutional Review Board/Ethics Committee of Seoul St. Mary’s Hospital, The Catholic University of Korea. The board waived the need for informed consent due to the retrospective nature of this study (KC22RASI0177).

### 2.2. Study Design and Patients

This study was a retrospective review of consecutive cases. Patients visiting the Department of Ophthalmology of Seoul St. Mary’s Hospital in Seoul, Korea, between January 2020 and January 2021 with a confirmed diagnosis of unilateral BRVO were included. The exclusion criteria were as follows: (1) a history of pre-existing retinal diseases or glaucoma; (2) high myopia (spherical equivalent > 5 diopters); and (3) significant media opacity.

The patients had undergone treatment with successful resolution of ME associated with BRVO. Treatments only included intravitreal anti-VEGF agents following a pro re nata (PRN) regimen, while some patients received no treatment. Retreatment criteria were as follows: CMT of over 300 µm measured by OCT, new or persistent cystoid retinal changes. All patients were monitored for twelve months after diagnosis, undergoing standardized dilated fundus examinations, including measurements of best-corrected visual acuity (BCVA). OCT examination at the final observation was performed in all patients.

### 2.3. Swept-Source Optical Coherence Tomography

**Figure 1:**
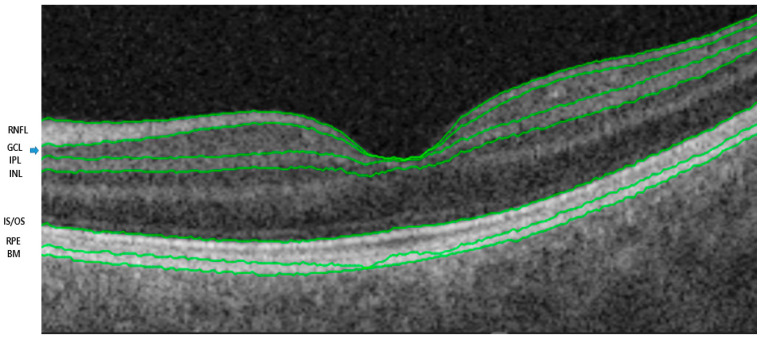
Automated segmentation of optical coherence image sets. RNFL: retinal nerve fiber layer; GCL: ganglion cell layer (blue arrow); IPL: inner plexiform layer; INL: inner nuclear layer; IS/OS: inner/outer segments of photoreceptors; RPE: retinal pigment epithelium; BM: Bruch’s membrane.

**Figure 2:**
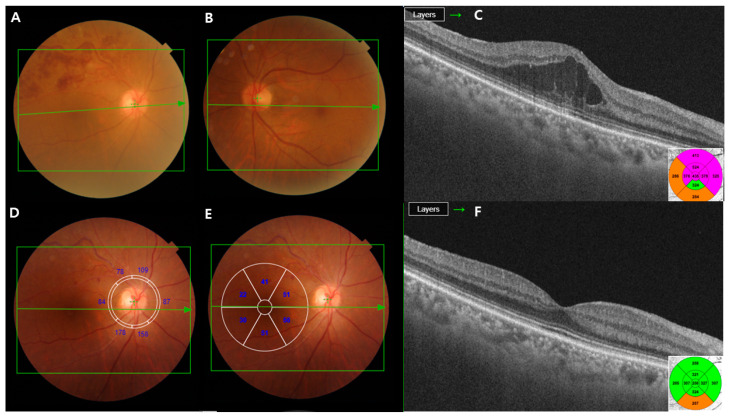
A 50-year-old man with branch retinal vein occlusion (BRVO) of the right eye. (**A**–**C**) The initial visit; the color fundus photograph shows retinal hemorrhage due to BRVO. The optical coherence tomography (OCT) image demonstrates macular edema (ME). (**D**,**E**) RNFL and ganglion cell layer thickness (GCL+) displayed in six macular sectors. Measured areas are displayed in six sectors (superonasal, nasal, inferonasal, inferotemporal, temporal, and superotemporal). (**F**) The OCT images reveal that ME almost resolves following the intravitreal injections of ranibizumab.

**Figure 3:**
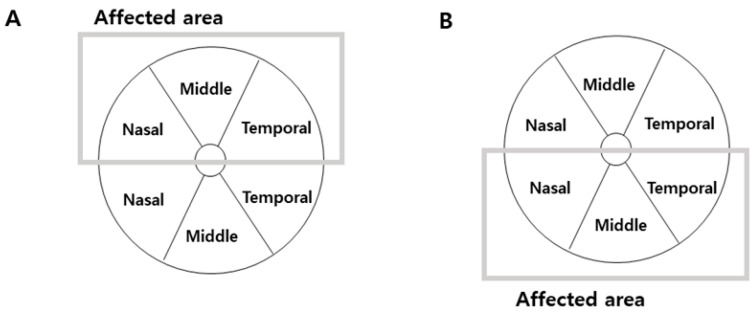
Schematic representation of the parameters automatically measured by Triton OCT in patients with BRVO. The ganglion cell layer thickness (GCL+ map) displayed in six macular sectors. (**A**) In the eyes with superior BRVO, we analyzed ganglion cell layer thickness in superior hemifield according to the affected area. (**B**) In the eyes with inferior BRVO, we also analyzed it in inferior hemifield according to the affected area.

The retinal and choroidal segments were measured using a deep-range imaging (DRI) Triton SS-OCT device (Topcon, Tokyo, Japan) with a wavelength of 1050 nm, 100,000 A-scans per second, and 8 mm axial and 20 mm transverse resolutions. This device provides images of better quality, allowing analysis of deeper layers than SD-OCT [18]. We used the three-dimensional wide protocol including a wide scanning range (12 × 9 mm^2^) that focuses on both the macular and peripapillary areas with the horizontal section passing directly through the center of the fovea. The SS-OCT performed automated segmentation of intraretinal boundaries (Figure 1). The CMT was measured from the inner limiting membrane (ILM) to the retinal pigment epithelium surface in the foveal region. The RNFL thickness was measured between the ILM and GCL boundaries at six peripapillary quadrants (temporal, superotemporal, superonasal, nasal, inferotemporal, and inferonasal). The GCL+ thickness map was measured between the RNFL and inner nuclear layer boundaries. GCL+ thickness measurements were also performed in six different sectors of the macular area (Figure 2). The sectors were reclassified according to the affected lesion (superior and inferior) (Figure 3). All scans were performed by the same experienced technician. The DRI Triton SS-OCT device has an image-quality scale to determine the signal strength. The images with a signal strength score < 55 were excluded.

### 2.4. Statistical Analysis

For statistical analyses, BCVA values were transformed into the logarithm of the minimum angle of resolution (logMAR) values. Paired t-tests were used to compare OCT parameters between BRVO eyes and unaffected eyes. Pearson correlation analysis was used to investigate the associations between OCT parameters and VA prognosis. Linear regression analysis was conducted to determine identifying factors related to VA as a dependent variable along with potential relative parameters. All statistical analyses were performed by using SPSS, version 22.0 software (IBM Corp., Armonk, NY, USA). A *p*-value of <0.05 was considered statistically significant.

## 3. Results

### 3.1. Baseline Characteristics

After excluding poor-quality OCT images, a total of 57 patients with ME secondary to treatment-naïve BRVO were enrolled. The patients’ mean age was 66.1 ± 10.42 years; there were 22 men and 35 women. Baseline visual acuity (VA) was 0.31 ± 0.18 logMAR, and the number of anti-VEGF injections was 3.20 ± 1.10. At the time of OCT, the mean logMAR VA improved to 0.29 with a resolution of ME in all eyes. The location of BRVO was found to be superior in 40 eyes (69%) and inferior in 17 eyes (31%). Table 1 shows the patient characteristics. The mean number of anti-VEGF injections over the 1-year follow-up period was 3.21 ± 1.10 (range: 0–6). No drug switching was observed during the study period. There was no difference in the mean number of anti-VEGF injections between the location of affected area (*p* = 0.67).

### 3.2. Optical Coherence Tomography Measurements

**Figure 4:**
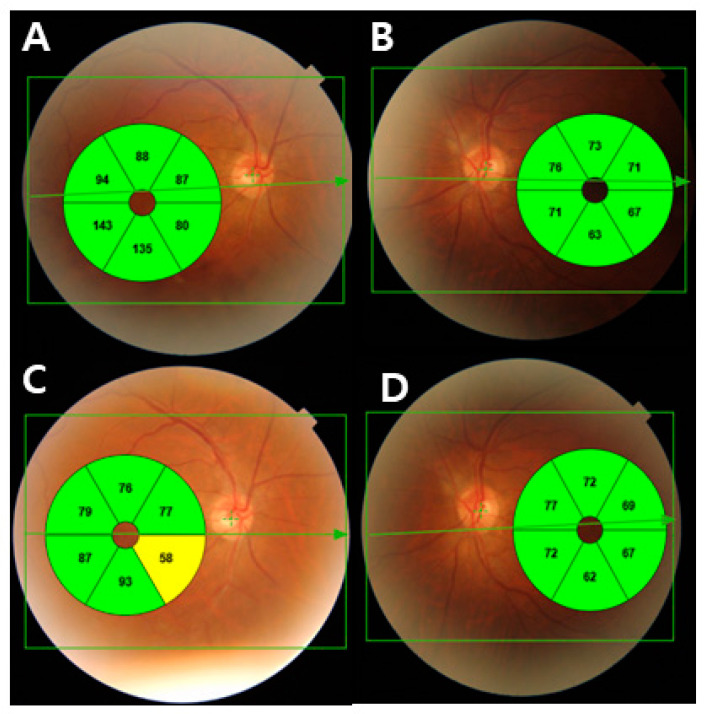
(**A**) Ganglion cell thickness map of a 67-year-old man with inferior branch retinal vein occlusion in his right eye. Due to macular edema, the ganglion cell thickness of affected sectors increased. (**B**) The fellow eye with normal ganglion cell thickness. (**C**) After the macular edema resolved, the nasal area of the affected area in the GCL thickness map demonstrated local thinning of the GCL thickness. (**D**) The fellow eye with normal ganglion cell thickness.

The mean CMT was 270.48 ± 32.7 μm, and the mean RNFL thickness was 105.46 ± 25.94 μm in BRVO eyes. The peripapillary RNFL thickness was analyzed by quadrants for BRVO eyes and unaffected eyes. Although the mean RNFL thickness was decreased in BRVO eyes compared to unaffected eyes, there was no significant difference between the two groups (*p* = 0.068). However, the temporal and nasal RNFL thicknesses were significantly different between BRVO eyes and unaffected eyes (*p* = 0.025 and *p* = 0.039, respectively) (Table 2). Among BRVO eyes, the affected quadrant had a significantly thinner GCL than the corresponding opposite unaffected eyes (*p* = 0.020). In particular, the nasal and middle areas of the affected quadrant demonstrated a significant decrease compared to unaffected eyes (*p* = 0.003 and *p* = 0.001, respectively) (Table 3, Figure 4). 

### 3.3. Correlation Analysis between Optical Coherence Tomography Parameters and Visual Acuity, and Number of Treatments

Correlation analysis showed that the average peripapillary RNFL thickness and CMT were not significantly correlated with VA (Table 4). The GCL thickness in the affected area showed a correlation with VA in the nasal and middle areas (*r* = −0.512, *p* = 0.003 and *r* = −0.537, *p* = 0.001, respectively). The average peripapillary RNFL thickness was significantly correlated with the number of injections (*r* = −0.286, *p* = 0.019). However, the CMT and GCL thickness in the affected area did not show a significant correlation with the number of treatments (Table 5). In the univariate and multivariate linear regression analyses for identifying factors related to VA, the GCL thickness of the nasal affected area showed a significant association (β = −0.09, *p* = 0.002).

## 4. Discussion

The specific findings of our study are as follows. First, although the average RFNL thickness was not significantly altered, the average GCL thickness was significantly thinner in BRVO eyes than in unaffected eyes. Second, the affected area in BRVO eyes had a lower GCL thickness than unaffected eyes. Third, there was a significant correlation between VA and GCL thicknesses at the nasal and middle areas.

Lee et al. previously described a reduction in the GCL thickness in eyes with BRVO [19]. To our knowledge, the present study is the first to examine GCL thickness as a significant predictor of VA in BRVO. The inner retinal layers, including the RGCs, are especially vulnerable to hypoxia, which causes alterations of the b-wave and oscillatory potential but not the a-wave in the flash electroretinogram [20]. Additionally, although the thickness of the outer retina is preserved in the retinal ischemia in animal models, the thickness of the inner layer is reduced [21]. Compromised foveal microvascular structures in eyes with BRVO can also affect the GCL thickness because they mainly affect the inner retina.

GCL thickness at the nasal area was significantly correlated with VA. We suggest that changes in the nasal area may reflect the changes in the inner retinal layers in the papillomacular bundle (PMB). The PMB is particularly more sensitive to ischemia than other areas. Pellegrin et al. described a case of metabolic optic neuropathy where the decreased macular vascular changes corresponded to areas of the PMB. They demonstrated vasculogenic changes not only at the peripapillary area but also at the macular area itself. Cho et al. also reported that the PMB was vulnerable to damage if accompanied by ischemic changes in BRAO or Purtscher-like retinopathy [22]. Further, because the nasal area was closer to vascular flow origins, i.e., the optic disc, than other areas, it might be more sensitive to flow pressure considering that vascular blood flow generates mechanical forces. However, further research is necessary to verify this hypothesis. To our best knowledge, this is also the first study on the changes in RNFL and GCL thicknesses in different areas.

SS-OCT is used in the study of various diseases, such as diabetic retinopathy, retinal vascular diseases, and age-related macular degeneration [23]. It is also used to identify subtle neurodegenerative changes in patients with BRVO. According to our results, prominent changes in GCL thickness could be found in the nasal area in BRVO eyes, and GCL thickness was related to the visual prognosis.

Our study also found that the thickness of the macular GCL and RNFL in BRVO eyes was significantly reduced compared with that of the controls and unaffected eyes. These quantitative parameters related to neurodegeneration may help to explain why some patients may have limited visual improvement despite complete resolution of ME in BRVO. Bra et al. reported that the ischemic area of the superficial capillary plexus was related to a thinning of the inner retina in OCT angiography [24]. This was related to the area of decreased GCL thickness. 

Yu et al. demonstrated that the inner retina was anoxic through their experiment, which seems to be related to providing a basis for the instability between the oxygen demand and consumption within the inner retina [25]. Because the choroid is not able to supply sufficient oxygen to the inner retina after retinal vascular injury, the area lacking blood flow would be related to the degree of visual dysfunction in diabetic retinopathy and BRVO. For example, glial cells (prominently in the RNFL) and RGCs have shown an increased expression of VEGF [26]. As a result, the excessive level of VEGF promotes breakdown of the blood–retinal barrier, and thus allows entry of circulatory harmful agents into the neuronal retina [27,28]. We hypothesize that mechanisms of vascular changes and neurodegeneration might be pathologically related, although further considerations are required to support this correlation.

Previous studies of laser-induced BRVO showed that venous tortuosity and tortuosity may appear immediately after venous obstruction [29,30]. These studies suggested that high venous pressure and increased vascular permeability cause retinal edema in the inner retinal layers, including the RNFL and GCL. Retinal edema can also be induced to reduce perfusion by compressing the capillaries [29,31]. Additionally, high intraocular levels of VEGF play a role in the development of ME in RVO [32,33]. These processes may be related to the pathogenesis of BRVO. However, comprehensive explanation of the vascular dysregulation related to the pathogenesis remains challenging.

The long-term visual losses are damage of photoreceptors and neuronal degeneration secondary to retinal hypoxia. Intraretinal fluid accumulation by itself causes Müller cell swelling and retinal degeneration. If the intraretinal edema persists, necrotic damage of Müller cells can cause irreversible neuronal degeneration [34,35]. For these reasons, the condition of photoreceptors and the ELM have been useful for predicting the visual outcome in BRVO eyes until now.

Although the association between outer retinal damage induced by ME and poor visual prognosis has been reported, the relationship between the inner retinal layers and VA has not been investigated [36]. In this study, we focused on the inner retinal layers, such as the peripapillary RNFL and macular GCL thickness. In the visual pathway, we believe that not only the integrity of the photoreceptors and ELM but also the states of the GCL and RNFL should be carefully investigated when deciding on treatment strategies in eyes with BRVO. Our results suggest that the automatic OCT-based thickness profile measurement can easily be applied in the prediction of VA.

We aimed to evaluate inner retinal damage that causes progressive visual loss. The thicknesses of the macular GCL and peripapillary RNFL decreased in BRVO eyes compared to unaffected eyes. Correlation analysis showed that the final VA was significantly correlated with the GCL thickness of the nasal and middle affected areas. The automatic measurement algorithm of the GCL provides the means to investigate the changes in ganglion cell loss in various sectors [37,38]. This parameter could be related to the location of local RGC loss in BRVO eyes. Ebneter et al. showed that the layers of the outer retina in the ischemic retinal thickness area are well preserved; however, the thickness of the inner retina layer is reduced in animal models of BRVO [21].

The potential toxicity of anti-VEGF injections in ganglion cells may affect the treatment outcome. Repeated suppression of neurotrophic cytokines after multiple anti-VEGF injections can be harmful to the RNFL [39,40]. Martinez-de-la-Casa et al. also reported that repeated anti-VEGF injections impaired the RNFL thickness because of the direct drug toxicity and fluctuations in the intraocular pressure in retinal diseases [41]. In our study, the number of intravitreal injections had a significantly weak correlation with the average RNFL thickness, although no correlation was found with the GCL thickness and mean CMT. 

There were a few limitations to our study. First, this is a cross-sectional observational study. A longitudinal study should be performed to explain the neuroretinal changes in BRVO. Second, there was an inherent sampling bias due to the retrospective nature of this study. We could not be certain of the time following resolution of macular edema at which the data were collected. In addition, further long-term prospective studies will provide data to better understand the neurodegenerative changes in the inner retina and their relationship with functional and anatomical results.

## 5. Conclusions

To summarize, during the follow-up period in patients with BRVO, there was a notable reduction in the average thickness of the GCL, and in the temporal and nasal areas of the RNFL in the affected area. The average peripapillary RNFL thickness was significantly correlated with the number of injections. In particular, final VA was significantly correlated with nasal and middle GCL thicknesses in the affected area. These findings suggest a potential association between inner retinal damage in ME secondary to BRVO and the visual prognosis.

## Figures and Tables

**Table 1:** Patient characteristics.

Characteristic	
Age (y)	66.1 ± 10.42
Sex, n	M:F = 22:35
Previous treatment, n	
Ranibizumab	18
Bevacizumab	30
Aflibercept	9
BCVA (logMAR)	0.31 ± 0.18
Duration (months)	16.78 ± 2.63
Central macular thickness (μm)	270.48 ± 32.7
Location of BRVO	Superior:inferior = 40:17

BCVA, best-corrected visual acuity; BRVO, branch retinal vein occlusion.

**Table 2:** Comparison of RNFL thickness parameters.

RNFL Thickness (μm)	BRVO Eyes	Unaffected Eyes	*p*-Value
Mean	105.46 ± 25.94	112.3 ± 18.36	0.068
Superonasal	97.65 ± 28.50	108.45 ± 36.65	0.389
Superotemporal	121.36 ± 44.29	125.36 ± 36.81	0.067
Inferonasal	119.57 ± 29.64	123.22 ± 26.51	0.088
Inferotemporal	135.63 ± 38.18	144.82 ± 33.59	0.974
Temporal	85.09 ± 21.83	85.80 ± 21.33	0.025 *
Nasal	75.69 ± 21.69	80.27 ± 23.70	0.039 *

RNFL, retinal nerve fiber layer; BRVO, branch retinal vein occlusion. * Statically significant at the *p* < 0.05.

**Table 3:** Comparison of GCL thickness parameters.

GCL Thickness (Affected Area)	BRVO Eyes	Unaffected Eyes	*p*-Value
Mean	63.43 ± 16.19	71.65 ± 16.94	0.020 *
Nasal	67.37 ± 17.55	78.03 ± 13.71	0.003 *
Middle	68.73 ± 19.23	73.34 ± 11.10	0.001 *
Temporal	56.37 ± 21.55	62.53 ± 13.69	0.470

GCL, ganglion cell layer; BRVO, branch retinal vein occlusion. * Statically significant at the *p* < 0.05

**Table 4:** Correlation of RNFL thickness, central macular thickness, and GCL thickness with visual acuity in BRVO eyes.

Parameter (μm)	Correlation	*p*-Value
Average RNFL thickness	−0.155	0.145
Central macular thickness	−0.073	0.238
GCL thickness (affected area)		
Nasal	−0.512	0.003 *
Middle	−0.537	0.001 *
Temporal	−0.221	0.172

RNFL, retinal nerve fiber layer; GCL, ganglion cell layer; BRVO, branch retinal vein occlusion. * Statically significant at the *p* < 0.05.

**Table 5:** Correlation of RNFL thickness, central macular thickness, and GCL thickness with the number of injections in BRVO eyes.

Parameter (μm)	Correlation	*p*-Value
Average RNFL thickness	−0.286	0.019 *
Central macular thickness	−0.069	0.314
GCL thickness (affected area)		
Nasal	−0.362	0.090
Middle	−0.085	0.304
Temporal	−0.061	0.348

BRVO, branch retinal vein occlusion; RNFL, retinal nerve fiber layer; GCL, ganglion cell layer. * Statically significant at the *p* < 0.05.

## Data Availability

The datasets analyzed during the current study are available from the corresponding authors on reasonable request.
